# Heterogeneity in prey distribution allows for higher food intake in planktivorous fish, particularly when hot

**DOI:** 10.1007/s00442-015-3485-1

**Published:** 2015-11-11

**Authors:** Z. Maciej Gliwicz, Piotr Maszczyk

**Affiliations:** Department of Hydrobiology, Biology & Chemistry Research Center, University of Warsaw, Al. Żwirki i Wigury 101, 02-089 Warsaw, Poland

**Keywords:** Capture rate, Learning, Global warming, Patch exploitation, Top–down cascade

## Abstract

When prey are scarce, planktivorous fish and other predators feeding on tiny prey should forage within prey-rich patches to attain a net food intake above the ambient mean food concentrations. If they can indeed locate prey-rich patches efficiently, then a patchy distribution of planktonic prey should lead to: (1) an increase in the overall per capita food intake, and (2) greater variability among predators in prey-capture rate due to differences in arrival times. Both phenomena were observed in 34 daily feeding sessions with a cohort of juvenile rudd held in twin experimental systems, each housing the same number of fish free to move in a loop of ten interconnected 200-L tanks. The fish were fed daily with equal numbers of planktonic prey (*Artemia* nauplii), offered either in a homogeneous or patchy distribution. To simulate low and high temperatures that represent potential global warming scenarios, the feeding protocol was replicated at 16, 21 and 26 °C, on each occasion following a 3-day period of fish acclimation. Up to 40–70 % of fish in the system with the patchy prey distribution assembled rapidly in the high-prey-density tank, the capture rate of first arrivals being up to 60 prey min^−1^ at 26 °C, orders of magnitude greater than that of latecomers. The overall capture rates were higher in the system with patchy prey, regardless of the temperature. At the highest temperature (26 °C), the fish located the high-prey-density tank in less than half the time taken at the lowest temperature (16 °C, *Q*_10_ > 2).

## Introduction

Resources with a patchy distribution are commonly assumed to be more profitable than those with a homogeneous distribution as ‘actively searching predators usually hunt for prey which is clumped or patchy in distribution’ (Krebs 1978; Stephens and Krebs 1986). There are only a few studies in the literature showing that the same quantity of resources would allow for a higher overall intake rate when patchily distributed. Currently, the best example of this was observed in a tactile foraging mallard (*Anas plathyrhynchos*), which was fed the same portions of cryptic prey (millet seeds mixed with sediment) at differently scaled heterogeneous distributions, with the foraging effort found to be concentrated within the patches of prey, and the overall intake rate higher at the coarse-scaled heterogeneous prey distribution than at the homogeneous one (Klaassen et al. 2006).

This should be expected in each typical predator foraging for small-bodied prey scattered in the vast space of the air (swifts or bats feeding on airborne insects) or in a virtually endless volume of water (fish feeding on planktonic crustaceans), i.e. in a predator that encounters prey in the three dimensions rather than in two dimensions, the latter being typical for those foraging in the terrestrial and benthic zones (Pawar et al. 2012). However, to our knowledge, no similar study has been performed on the most typical predator feeding on tiny prey, the planktivorous fish, despite so much already known about patch exploitation by fish. This was first suggested by Lasker (1975), who demonstrated that anchovy larvae starved when their planktonic prey was not patchily distributed. Also, we know of no research addressing the question of whether a higher capture rate is assured in a patchy distribution of prey compared with that in a homogeneous distribution of the same number of prey, and its implications for understanding the reasons underlying patch formation in zooplankton prey as an anti-predation refuge (Gliwicz et al. 2013).

The first aim of this study was to test the hypothesis that prey-capture rates for planktivorous fish may increase when they feed in an environment where prey have a patchy distribution, compared to feeding when prey is homogeneously distributed, and to determine whether the exploitation of prey patches follows one of the known models of patch exploitation. A patchy distribution of prey invokes the question of inequality of capture rates even when it is assumed that predators both follow the principle of the marginal value theorem [MVT; the individual-centered approach of Charnov (1976), and Krebs (1978)] to leave the patch before the prey has been decimated, and also observe the rules of ideal free distribution [IFD; the population approach of Fretwell and Lucas (1970)] to distribute themselves in correct proportions within and outside the patch of prey. However, this distribution and subsequent movement away from the patch require time, thus supporting the theory that there must always be an initial predator entering an unexploited patch of prey before any others, giving it the rare opportunity to feed at a prey abundance with the theoretically highest intake rate. The intake rate of the next predator joining the feast would be much lower, and the prey density found by those following is reduced with increasing speed as more and more predators feed within the patch. This aspect is rarely considered in studies on the interface between planktivorous fish and their zooplankton prey, and so the second aim of this study was to test the hypothesis that patchily distributed prey leads to variability in individual capture rate. This variability may theoretically lead to a broader spectrum of individual fitness when a chance, earlier arrival to the patch of prey might allow an accidental fish both a greater intake rate and better orientation at the time of the next feeding opportunity.

Another unexplored phenomenon is the strong effect temperature has on the rapidity of locating prey patches. Changes in temperature may affect the swiftness of fish searching for patches of prey, as well as influencing their ability to learn (McNamara and Houston 1987) and memorize (Milinski 1994; Webster and Hart 2006) the spatial structure of prey distribution. The third goal of this study was to quantify the effects of increased water temperatures on prey acquisition, in particular, to what extent the rapidity of patch exploitation (which depends on the functional and numerical responses of predators to patchy prey distribution) would be amplified by the increased temperature caused by global warming, and whether such amplification would be higher than expected from the *Q*_10_ = 2 assumption (i.e., that the fish metabolic rate is doubled as the temperature increases by 10 °C). This would result in strengthening the top–down impacts of fish predation on zooplankton and phytoplankton. The expectation that such predation is amplified by global warming is supported by the likelihood that the energy investment for post-capture accelerations is reduced at higher temperatures due to lower water viscosity (Gliwicz et al. 2013) and that reduced energy is required at higher temperatures for warming the neural system of fish, particularly their brains and visual sensors (Sepulveda et al. 2007) vital for memory, learning, and obtaining and processing sensory information needed for continued prey acquisition.

## Materials and methods

### The experiments

Two groups of fish (rudd, *Scardinius erythropthalmus*) chosen at random, were exposed to the same number of prey but with the latter distributed either uniformly or patchily, crossed with three different temperatures (16, 21 and 26 °C). The range of 16–26 °C was chosen based on the assumption that a typical planktivorous juvenile cyprinid spends most of its active life in summer between the surface (the twilight foraging for zooplankton at temperatures approaching 26 °C on hot and windless days, likely to become more common due to global warming) and the upper metalimnion (daytime hiding in darker and cooler water, rarely below the depth at which the temperature is 16 °C). The rudd is a typical planktivorous predator and we used juveniles of 40–50 mm in length. All fish were the offspring of ten females and ten males, hatched in the laboratory in April 2013. After preliminary trials, we chose *Artemia* nauplii as the prey, hatched daily from dried cysts (Sanders Brine Shrimp) from the fishless Great Salt Lake, Utah, USA. The nauplii survived for longer than 24 h in the freshwater experimental system and were highly motile when uneaten prey was removed from the system at the end of each 8- to 12-min feeding session. *Artemia* are a reliable prey source because they are easy to culture in a laboratory setting and representative in size and mass of the small cladocerans, which are the typical prey item for cyprinid fish, such as rudd or roach (*Rutilus rutilus* L.), and are commonly available to planktivorous fish in temperate lakes in densities between 1 and 50 individuals L^−1^ (Gliwicz 2001), which were the densities of *Artemia* prey used in this study. Preliminary experiments revealed an optimal spectrum and intensity of light during feeding sessions equal to 1 μmol m^−2^ s^−1^ of photosynthetically active radiation, corresponding to the light levels in a mesotrophic lake measured on the evening of a sunny day (1800 hours) in July at 1-m depth Gliwicz 2001), and an optimum duration of feeding sessions lasting from 2 to 15 min, depending on temperature, which left 10–40 % of prey uneaten.

**Fig. 1:**
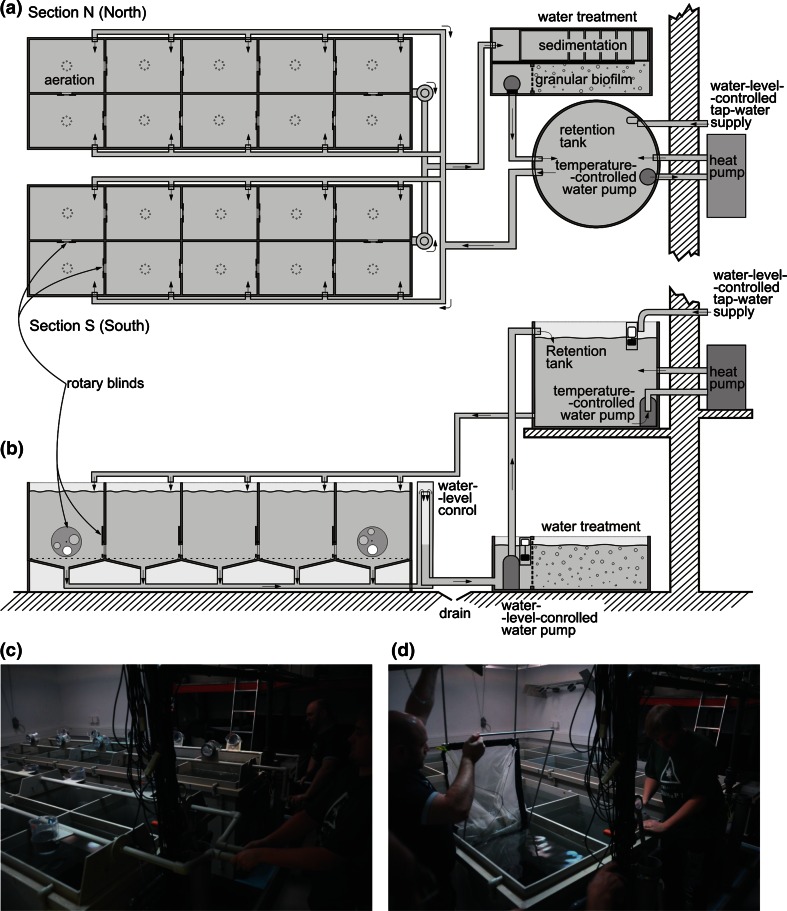
The twin experimental system [sections north (*N*) and south (*S*)] with their shared mechanical and biological purification plant, and the 1000-L reservoir (retention) tank allowing constant water circulation at a rate set by the flow controller in each tank’s inflow (AQUA TECH, Poland). Each of the two sections houses the same number of fish (50–120) in the ten 200-L tanks interconnected into a loop to allow two-way unrestricted movement of fish in their quest for the most rewarding site (tank) with the highest density of *Daphnia* or *Artemia* prey. One section contains either heterogeneous or patchy prey distribution, while the other section contains the same number of prey at a homogeneous distribution. **a** Aerial view, **b** lateral view, and **c** a wide-angle view at the start of the daily feeding session, when the prepared portions of live prey are simultaneously flushed into the tanks of each section with the assistance of four feeders. **d** A view at the end of the feeding session when the fish have been herded into one (already sampled in the dark) tank to allow removal of the remaining prey from the other tanks in each section using a plankton net in a steel frame that exactly fits the tank’s cross-section dimensions. One of the two pairs of feeders is seen resting on the wall. Note that the aeration system and its air pump have been omitted for clarity and the video cameras have been put aside to allow a clear view of the feeders

The same group of 220 fish of similar fresh mass (mean ± SE, 1.1 ± 0.3 g) and length (5.4 ± 0.5 mm) was used in the three successive experiments (1, 2 and 3), each carried out at 16, 21 and 26 °C (Table 1). All of the fish were released into a twin tank system; half were released into the north section and the remainder was released into the south section (Fig. 1a, b). All the experiments started after a 2-week acclimation period, during which *Artemia* were distributed to both sections each afternoon to simulate either patchy distribution in one section (91 % of prey in one of the ten tanks treated as a patch of prey, and 1 % in each of the remaining nine tanks) and homogeneous prey distribution in the other section (10 % of prey in each of the ten tanks). The prey was thoroughly mixed in each tank of both sections by rotating the feeders (Fig. 1c; see “Prey release into the system” section). This initial period eliminated the effect of fish learning the three-dimensional structure of the set up and the patchy distribution of the prey. These effects were seen to be strongest in the first week of the fishes’ presence in the systems, and evidently faded away during the second week of acclimation to become undetectable in the course of the three subsequent experiments. For all of the experiments (1–3) at each of the three temperatures, the fish were offered the same portions of prey at either the homogeneous distribution (10 % of the portion in each of the ten tanks) or the patchy distribution (91 % of the portion in one tank and 1 % in each of the nine remaining tanks) during afternoon feeding sessions. Experiments 1 and 3 had patchy prey distributions in the south section with homogeneous prey in the north section, whereas experiment 2 had patchy prey distributions in the north section with homogeneous prey distribution in the south section. Due to the initial 2-week acclimation of all experimental fish preceding experiment 1, there was no need to start each experiment with a new 2-week acclimation period.

**Table 1:** Details of the three experiments and their core results describing fish-capture rates at the same initial numbers of *Artemia* prey with homogeneous and patchy distributions in the twin experimental systems with initial prey density of 91 % in the high-prey-density tank and 1 % in each of the remaining nine tanks of the section with patchy prey distribution, and 10 % in each of all ten tanks of the section with homogeneous prey distribution

Starting date	No. of successive feeding sessions	Temperature	Duration of feeding session	Initial prey density in high-prey-density tank	Percentage of captured prey in the system with prey		Difference between the two systems
		°C	Minimum	Individuals L^−1^	Homogeneous distribution	Patchy distribution	%
Experiment 1							
1 August	3	16	8–12	24.3–25.7	82.3–88.8	85.2–94.6	0.9–10.1
6 August	4	21	8–12	16.4–23.2	71.3–86.6	80.0–93.3	7.0–17.1
12 August	3	26	8–10	17.3–32.2	88.0–91.3	92.2–93.4	2.2–4.6
Experiment 2							
17 September	4	16	8–12	10.0–26.8	33.3–78.8	55.3–93.0	15.3–39.8
11 September	4	21	8–12	14.4–23.9	71.6–78.3	84.7–94.6	7.6–22.7
04 September	4	26	8–10	19.8–27.6	70.4–84.1	77.0–94.6	7.9–20.0
Experiment 3							
26 October	4	16	8–12	20.0–25.4	65.4–73.9	84.5–89.1	12.6–26.6
3 November	4	21	8–12	17.0–22.3	54.9–83.4	65.3–94.6	5.8–33.9
10 November	4	26	8–10	13.5–31.3	32.8–75.9	35.6–92.5	8.0–26.9

Note that the sequence of temperatures was inverted between subsequent experiments to minimize the number and duration of enforced fish thermal acclimations (experiments 1 and 3 were started at 26 °C but experiment 2 at 16 °C), and that 12 additional shorter (2- to 6-min) sessions (four sessions at each of the three temperatures) were performed within experiment 3 to measure the rate of prey density decline at the beginning of the feeding session. The time limitation of temporarily housing 10,000 *nauplii* in 4-L calibration flask allowed only two to three corrections to be made by subsequently adding or removing prey, leading to fluctuations in the initial prey numbers (but not the proportions) offered to fish

All of the experiments had four afternoon feeding sessions at 21 °C, and three to four sessions at 16 and 26 °C. The sequence of temperatures was inverted in experiment 2, whereby the first feeding session started at the temperature of the last session of the preceding experiment (Table 1). Fish were fed with the same numbers and the same distribution of *Artemia* prey also in the course of the 3-day intervals used for the water and air temperature changes, and for the fishes’ thermal acclimation between the sessions at the different temperatures. At the end of experiment 3, the experimental fish were anaesthetized in a solution of 2-phenoxyethanol in water [0.45 g L^−1^; Myszkowski et al. (2002)] to measure their final fresh weight and length, which was nearly the same as the initial weight (respectively, 1.2 ± 0.3 g, and 5.5 ± 0.6 mm), and then returned to the stock tank.

The variability in the duration of each feeding session (8–12 min; Table 1) resulted from the different times needed to stop the further increase in the number of fish in the high-density tank of the section with patchy prey. The number of fish in this tank was continuously monitored, and determined as the difference in the number of fish entering the tank and the number of fish leaving the tank, clearly visible via the video system (see “The experimental system” section).

Experiment 3 was extended with three to four additional feeding sessions (2, 4 and 6 min) at each temperature in an attempt to determine the rate of prey decline, and thus the capture rate, in the first minutes of the feeding session when the prey was most abundant. This lengthened the duration of exposure at each temperature by an extra 4 days (Table 1).

Care was taken to ensure that all tanks in each section were free of past or present signals by carefully replicating light levels, the timing of prey delivery, duration of feeding session within the range of 8–12 min, and changing the position of the high-prey-density tank daily by moving it clockwise by three subsequent tanks. Constant water circulation ensured that water in the two sections was mixed when in the water treatment plant and retention tank. In more than 2 years of its use, the system remains free of fish parasites.

### The experimental system

The twin experimental systems (made of light polyester-glass fibre laminate by AQUA TECH) resemble the system used in our earlier studies on patch exploitation and prey selection by planktivorous fish (Gliwicz et al. 2013; Maszczyk and Gliwicz 2014), except that the volume of each section has been reduced (from 8 to 2 m^3^), the horizontal dimensions reduced (from 600 × 300 to 320 × 100 cm), and the arrangement of eight circular 1-m^3^ tanks (122 cm in diameter) interconnected by pipes (of 15 cm diameter) has been replaced by ten rectangular (60 × 40 cm) 200-L tanks, sharing two sides with the neighbouring tanks, each of which has an opening. These openings have adjustable apertures of 8, 12 or 15 cm in diameter controlled by a rotary blind closure to allow isolation of each tank when the blinds are fully closed and free movement of fish throughout the entire section of ten interconnected tanks when the blinds are opened. The two identical sections are accommodated in a room of 60 m^2^ and 4 m in height (Fig. 1).

Infrared video cameras (with their objectives submerged 1 cm below the surface) were used to detect fish moving between section compartments via openings with rotary blinds placed close to the bottom of each tank. This bottom was formed by a replaceable steel plate with 20 apertures of 1 mm diameter in the centre to allow both the outflow of waste water and the inflow of air from a central air pump, which produced a stream of bubbles that gave sufficient water saturation with oxygen and mixed the prey within each of the ten tanks in each section.

The volume of the entire system was 5.5 m^3^, with water constantly pumped up from the water treatment tank to the retention tank. The spillage caused by prey sampling was automatically compensated by temporary inflows from the mains to the retention tank of 1000 L capacity supported on a steel frame with the water’s surface 2.2 m above the floor to ensure a constant gravity-assisted flow to each of the ten tanks in both sections (2 m^3^ total capacity each, 200 L in each tank). The individual water level in each tank was controlled by a siphon cascade, with the waste water flowing by gravity into a water treatment tank of 500 L capacity composed of a sedimentation chamber, biological purification system with granular biofilm, and a pump compartment (Fig. 1a, b). The cleaned water was then transferred using a submerged electric pump back to the retention tank. The retention tank was connected by a separate water circuit using another pump to a reversible heat pump (Airland HP Booster Technology, China) that either heated or cooled the water before it was returned by gravity to all 20 tanks of the twin sections. To allow automatic replacement of the water lost each day during sampling, the reservoir tank was also connected to the main water supply via a valve regulating the water level in this tank. Losses by evaporation were considerably reduced by automatic air cooling with an air conditioning system and heating by an efficient air heater (EWT model CFH120, 12 kW) directed towards the floor between the two sections. This effectively controlled the air temperature within narrow limits (±0.2 °C) to maintain the three water temperatures (16, 21 or 26 °C) used in this study. The rate of temperature change over each of the 3-day intervals between experiments was 1.33 °C per day.

The illumination system was suspended from the ceiling above the twin sections. It was composed of ten lamps, each comprising 160 diodes: 40 diodes for each of the four colours blue, green, orange and red. Each lamp shone from an elevation of 3 m through a semi-transparent Plexiglas diffusion plate onto the middle of the wall between the two parallel tanks in each of the five pairs of tanks connected into a loop in both of the sections (Fig. 1a, b). This method of illumination gave a similar light intensity in the tanks, as was confirmed by LI-COR (model 189) readings taken at the bottom of each tank. The light spectrum was always equivalent to natural solar radiation (at other spectral ranges tested in the preliminary experiments, the capture rates were slightly lower than in the full spectrum of light). The light intensity at 0.5 m depth was 0.8 ± 0.2 μmol m^−2^ s^−1^ throughout the feeding session. This preceded a gradual dusk decline, then nighttime with a reduced light intensity, followed by a gradual increase in the morning to the full daytime light level.

### Prey release into the sections

Two feeders composed of five 1-L cups nested in a revolving frame were used to deliver the appropriate amounts of prey to each section. Each portion was flushed from a single cup to one of the five tanks served by one feeder, with the other five tanks in the section served by the other feeder, the two feeders for each section operated by one person turning them at the same time through two revolutions to ensure that all prey items were flushed simultaneously and completely from the cups (Fig. 1c). All four feeders were returned to their rests after all prey portions had been delivered (Fig. 1d), and the 20 cups were taken away to the lab until the next feeding session, leaving the fish alone in the experimental room.

Staff conducting the experiment gathered by the computer monitor next door to observe (and record for fish speed estimates) live video of fish activity in two tanks in each section, one of which was always the high-density tank in the section with patchy distribution, containing 91 % of the prey at an initial density of between 13 and 31 individuals L^−1^ (Table 1). This patch of prey was created in a different tank each evening by moving its location three tanks forward around the section, i.e. from tanks 1 to 4, next to tank 7, then tanks 10, 3 etc.

The 20 prey portions were prepared immediately before each feeding session by introducing the required number of prey into a 5-L calibrated plastic brand jar. This was achieved following enumeration of the prey in small sub-samples using a dissecting microscope, in order to obtain the correct number of prey for both sections plus three 1 % samples that were preserved for later detailed counts. The content of the jar was divided volumetrically into two equal portions, one for each of the sections. Each portion was then divided into either ten equal portions into ten cups (for the section with the homogeneous prey) or nine 1 % portions for nine cups, leaving the remaining 91 % of the volume to fill the 10th cup (for the section with the patchy prey). These smaller portions were taken using a Stempel pipette (Wildlife Supply) as the mother sample was being mixed.

### Enumerating remaining prey

Immediately before the end of a feeding session, the visible light was switched off and staff equipped with infrared goggles entered the experimental room through an illumination lock/chamber to shut all inter-tank windows, count the fish accumulated in the high-prey-density tank of the section with patchy prey distribution (e.g., tank 7) while transferring them to the neighbouring tank (tank 8) which had been first sampled for the remaining prey, transfer all fish of the section to this tank (tank 8), do exactly the same in the respective tanks (e.g., tanks 7 and 8) in the section with homogeneous prey distribution, and—after switching the lights on—sample all nine remaining tanks of each of the two sections. The brief, high density of fish (up to 0.5 fish L^−1^) in one of the tanks (tank 8) was assumed to be below the critical density that might have caused interference competition in roach of a similar size tested in a similar experimental system (Maszczyk et al. 2014).

The entire 200-L volume of each tank was sieved twice through special 0.16-mm-mesh plankton net held in a steel rectangular frame (595 × 395 mm) with two foldable handles (395 × 600 mm) mounted on the sides of the frame (Fig. 1d). The retained material was transferred into bottles, fixed with 4 % formaldehyde, and stored for later enumeration under a dissecting microscope. Then, all inter-tank windows were opened to allow the fish free movement around the ten tanks in each section. The difference between the number of prey introduced into each tank and the number remaining in that tank at the end of a feeding session was assumed to represent the number of prey captured by the fish feeding in the one high-density tank and the nine low-density tanks of one section, and in all ten tanks of the other section in the time of the 10- to 12-min feeding session.

### Estimating the number of fish in the high-prey-density tank

The final number of fish in the high-prey-density tank counted at the end of each feeding session immediately after the illumination was switched off, combined with the number of fish entering and leaving the high-prey-density tank through one of the two connecting gates registered on the video recordings, allowed back calculation to be made of the number of fish at any particular time point. At the start of each feeding session, this number roughly corresponded to a less precise count of the initial number of fish in the high-prey-density tank (some fish close to the water’s surface were not recorded by the video cameras).

### Estimating the speed of fish movement

For all experiments, swimming speeds (centimetres per second) were measured using archived video images by comparing two successive images and measuring the distance that an individual fish travelled in the bottom 20 cm of the tank (using a scale marked at the bottom of the tank) and dividing by the elapsed time. The distance travelled from non-linear movements was estimated after the tracks were straightened out. All these calculations were repeated for individual fish for each experimental treatment (up to 133, 177, and 106 individuals in the tank with, respectively, patchy high, patchy low, and homogeneous distribution).

### Calculation of capture rates in subsequent fractions of a feeding session

The additional, shorter (2- to 6-min) sessions allowed calculations to be made of capture rates in each 2-min interval of the feeding session. This was possible due to the continuous video recording of fish entering and leaving the tank containing the patch of prey, and by assuming that the number of fish in the remaining nine tanks of the section with patchy prey was the same, as was also assumed for all ten tanks of the section with the homogeneous prey. This assumption was necessary as prey samples were pooled for the nine low-density tanks of the section with patchy prey, and for all ten tanks of the section with homogeneous prey. There was some variability in the number of fish present in the high-prey-density tank resulting from the different rates at which fish distributed themselves in relation to prey density, so the capture rate had to be calculated as the number of prey eliminated divided by the mean number of fish present in the tank for a given period of time (2 min).

### Data analysis and statistical methods

Prior to the main data analysis that would allow the testing of the three main hypotheses, a regression analysis was performed as a pre-test of the probability of learning by the experimental fish throughout the entire 70 days of the experimental period, from the first feeding session of experiment 1 to the last session of experiment 3, with the day of experiment as the independent variable (to determine if there was a temporal trend). The separate analysis of regression was performed for each of the three temperatures treated either separately or jointly for each of the two parameters: the change of the mean capture rate in each section (patchy or homogeneous prey distribution) throughout the entire experimental period, and the time needed for 25 % of fish to congregate in the high-prey-density tank of the section with patchy prey distribution during a feeding session.

In order to test the hypotheses that patchily-distributed zooplankton prey secures higher overall capture rates than the same quantity of prey in a homogeneous distribution, and that this effect is more apparent at higher temperatures, a two-way ANOVA was used with prey distribution and temperature as subject factors. This statistical method was also used to test the effect of temperature on the overall capture rate.

In order to test the hypothesis that patchily distributed prey leads to increased variability in individual capture rate, the coefficient of variation (% of the mean) was compared: (1) for fish in the high-prey-density tank, and fish in the nine remaining tanks of the section with patchy prey; and (2) for all fish from the section with patchy, and all fish from the section with homogeneous prey. In order to test the hypothesis that increased variability in the individual capture rate of patchily distributed prey is more apparent at higher temperatures (higher temperature being responsible for increased variability in capture rate), this pairwise comparison was performed for each of the three temperature variants.

In order to examine the effects of temperature and prey distribution on the variability in capture rate, an additional analysis was made of the mobility of fish in their search for the most profitable site. This was done using a video recording of the gradual change in the number of fish in the high-prey-density tank as well as the number of fish entering and leaving the tank each minute. This, combined with the additional short-lasting feeding sessions, provided an estimate of the number of *Artemia* prey removed by fish within the first 1, 3, 5, 6.5, 7.5 and 10 min at 21 °C and within similar time periods at 16 and 26 °C. These data, combined with fish video counts, were used to determine average capture rates at different phases of prey exploitation. The video recording was also used to determine the individual speed of fish foraging for *Artemia* prey at each of the three temperatures at each of the three initial prey density levels: in the high-prey-density tank, in the nine remaining tanks of the section with patchy prey distribution, and in all of the ten tanks of the section with homogeneous prey. Although the speed of fish foraging for *Artemia* prey has previously been found to be highly variable (range of 1–20 cm s^−1^), this variability was much reduced at a high density of *Artemia* prey, with nearly half of the fish in the high-prey-density tank found to have a swimming velocity below 7 cm s^−1^ (Table 2). Such a swimming speed is typical for rudd moving to a prey item, as observed in video recordings of rudd during experiments on their reaction distances at different light levels (Babkiewicz and Kumar, unpublished data). This speed of 7 cm s^−1^ is assumed to be the borderline between the two modes of foraging in rudd, low-speed harvesting and high-speed searching, with harvesting speed useful only at prey densities that are high enough for fish to see the next prey within the reaction field volume at the time the previous prey is being captured (Gliwicz et al. 2013).

**Table 2:** Mean (±1 SD), range, and proportion (%) of speed below 6 cm s^−1^ in the section with patchy prey distribution in the high-prey-density tank (with the initial 91 % of prey) and in the remaining nine tanks (with 1 % of the prey added to each), and in the section with homogeneous prey distribution (with 10 % of prey added to each of ten tanks), at three different temperatures (16, 21 and 26 °C)

Temperature	Prey distribution	Patchy high	Patchy low	Homogeneous
	Initial prey in the tank (%)	91	1	10
16 °C	Number of observations (*n*)	133	167	58
	Mean (cm s^−1^)	8.47	10.78	8.98
	SD (cm s^−1^)	5.13	4.89	4.84
	Range (cm s^−1^)	1–20	1–21	1–19
	Below 7 cm s^−1^ (%)	51.1	26.3	46.6
21 °C	Number of observations (*n*)	100	177	106
	Mean (cm s^−1^)	8.55	11.99	9.90
	SD (cm s^−1^)	4.94	7.10	5.28
	Range (cm s^−1^)	1–21	1–32	1–23
	Below 7 cm s^−1^ (%)	44.7	28.8	35.8
26 °C	Number of observations	75	96	47
	Mean (cm s^−1^)	9.24	14.58	8.66
	SD (cm s^−1^)	6.32	7.51	5.37
	Range (cm s^−1^)	1–29	1–33	1–25
	Below 7 cm s^−1^ (%)	50.1	13.5	48.9

Although most of the results focusing on fish movements in the two sections are of a purely descriptive nature, there are two exceptions. One is the capture rate in the initial 2 min of the feeding session, and the other is the mean swimming speed of fish foraging at each of the three levels of prey density for the entire feeding session, each estimated from the number of prey eliminated in a given tank or the entire section. The differences between the two sections were examined using the two-way ANOVA test with prey density and temperature as the two subject factors.

Additional analyses were made of the effect of temperature on the time needed for 15, 20 and 25 % of fish to congregate in a patch of prey, and the number of fish entering and leaving the high-prey-density tank in the first minutes of the feeding session, as well as the number of prey eliminated from the high-prey density tank in the first 2 min, all tested by one-way ANOVA. In order to quantify the strength of fish behavioural responses to the temperature increase, *Q*_10_ values were calculated to see if the impact of increased temperature is doubled as the temperature increases by 10 °C (*Q*_10_ = 2) or if the effect is actually stronger than expected (*Q*_10_ > 2). Tukey’s honest significant difference post hoc test was used to complete all ANOVA analyses, not exclusively those with two-way but also those with one-way ANOVA, as there were always three experimental temperature levels. When comparing experimental data from the three different prey densities in the two sections (high- and low-prey-density tanks in the section with patchy prey, and intermediate prey density in the section with homogeneous prey distribution), it was assumed that the foraging behaviour of fish was not dependent on the prey densities in other tanks.

## Results

### Learning ability: a pre-test

The analyses of regression lines for the consecutive feeding sessions throughout the entire 70 days of the experimental period from the first feeding session of experiment 1 to the last session of experiment 3 (Table 1) did not reveal any significant change in either the mean capture rate of fish foraging in any of the two sections, or in the time needed for 25 % of fish to congregate in the high-prey-density tank of the section with patchy prey distribution. The only marginal difference was detected at *P* = 0.0385 (*F*_1,8_ ≤ 2.8) in the slope of the regression line for the time needed for 25 % of fish to congregate in the high-prey-density tank of the section with patchy prey distribution, but only at 26 °C and not at the other temperatures. The lack of significance in the majority of cases suggests an absence of an apparent learning ability in experimental rudd from one feeding session to another, or is a result of the earlier fortnight pre-experimental experience, but does not rule out the possibility that the fish may instead possess long-term memory.

### Overall capture rate in patchy and homogeneous prey distributions

**Fig. 2:**
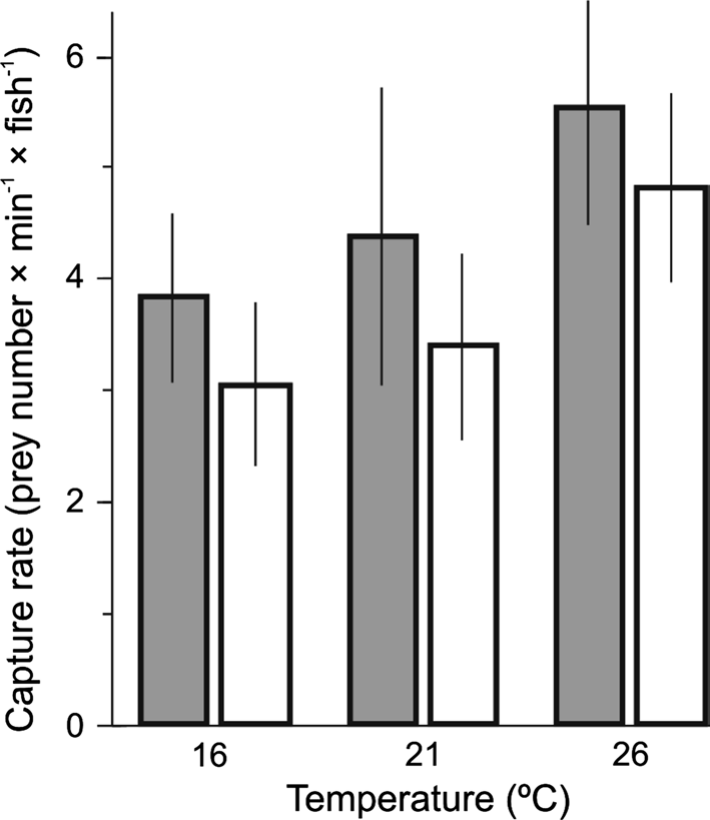
Mean (±1 SE) overall capture rate per individual fish throughout the feeding session in the section with the patchy prey distribution (*shaded bars*, *n* = 40) and in the section with the homogeneous prey distribution (*unshaded bars*, *n* = 40) at three different temperatures (16, 21 and 26 °C)

**Fig. 3:**
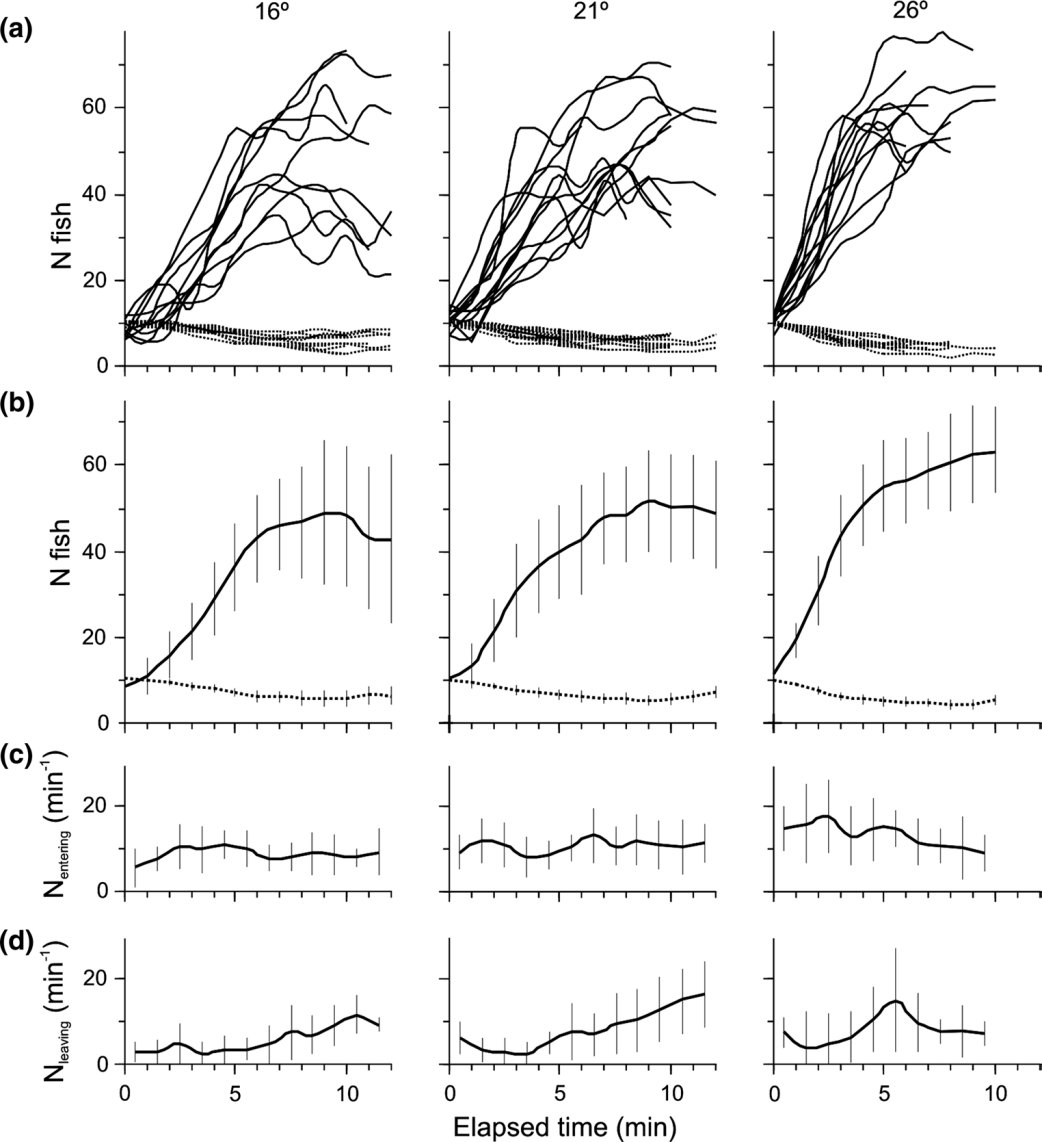
Numerical response of fish to patchy prey distribution at three different temperatures (16, 21 and 26 °C). **a** The number of fish (% of the total number of fish in the section with patchy prey distribution) in the high-prey-density tank (with 91 % of the prey added to the section; *solid lines*) and in each of the nine remaining tanks (each with 1 % of the prey added; *dashed lines*) each minute, each line for one of the ten replicate feeding sessions; **b** the same as the best fitted line for the mean (±1 SE) from ten feeding sessions in the high-prey-density tank (respectively, *solid* and *dashed line*); **c** the experimental data on the number of fish entering the high-prey-density tank each minute (mean ± 1 SE, *n* = 10); **d** the experimental data on the number of fish exiting the high-prey-density tank each minute (mean ± 1 SE, *n* = 10)

**Fig. 4:**
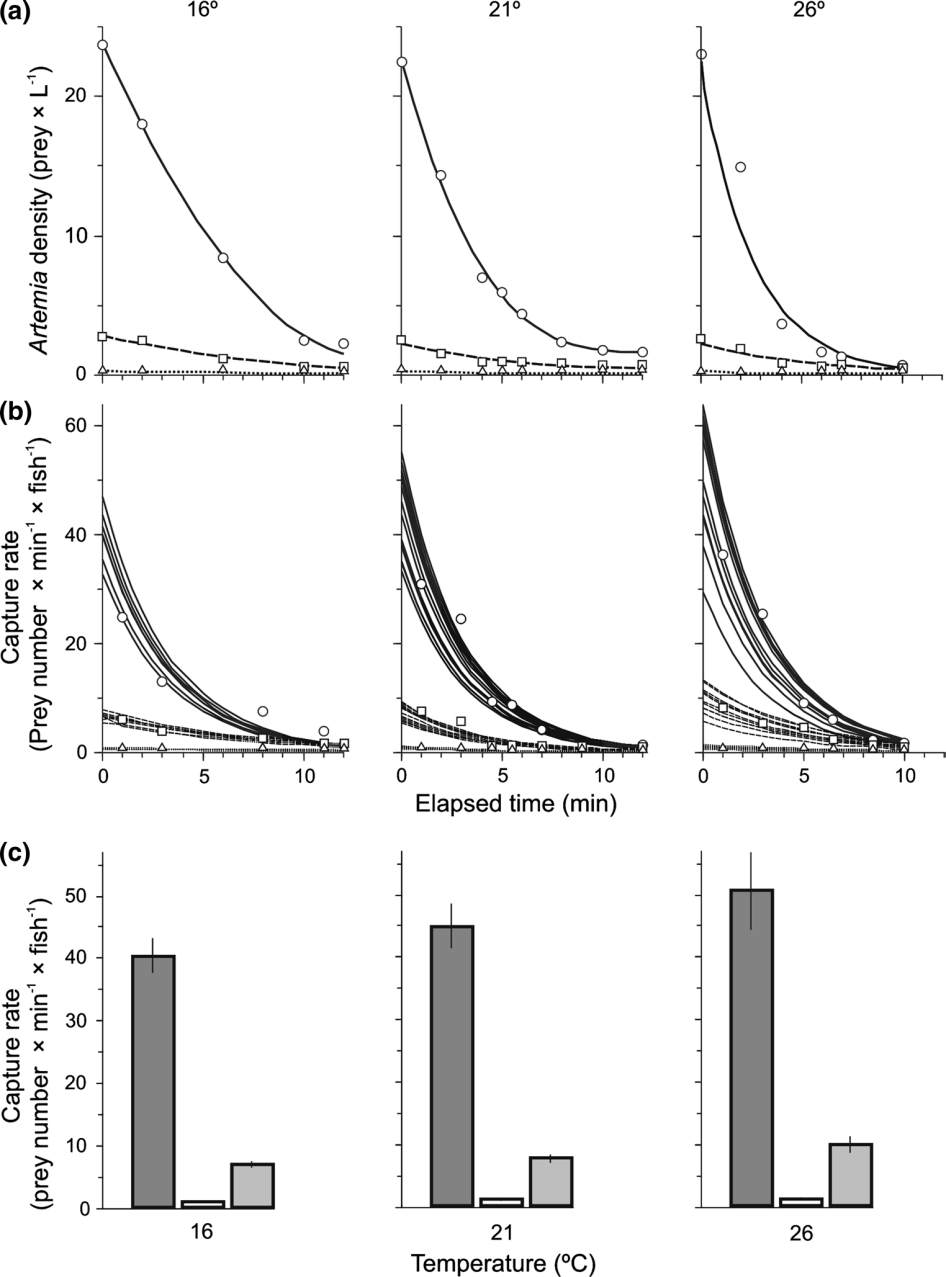
Prey density decline (**a**) and change in mean capture rate per individual fish throughout the feeding session (**b**) and in its first 2 min (**c**), each shown within the patch of prey in the section with patchy prey distribution (in the tank with 91 % of the prey added; *solid lines* and *columns on the left*) and outside the patch (in the remaining nine tanks with 1 % of the prey added to each; *dotted lines* and *columns in the centre*), as well as in the section with homogeneous prey distribution (10 % of prey added to each of ten tanks; *dashed lines* and *bars on the right*) at three different temperatures, 16 °C (*n* = 5), 21 °C (*n* = 8) and 26 °C (*n* = 6), as calculated from the best-fitted exponential lines based on the additional preliminary experiments with feeding sessions lasting for different periods from 2 to 15 min (data from these sessions shown as, respectively, *circles*, *squares* and *triangles* in **a** and **b**)

The difference in the overall capture rate between the two sections was immediately apparent as the different percentage of captured prey shows (Table 1). The mean overall capture rate of fish throughout all the feeding sessions was found to be significantly greater in patchy prey distribution than in homogeneous prey distribution of exactly the same number of prey added to each of the two sections at each feeding session (Fig. 2; *P* = 0.042, *F*_1,83_ = 4.3), and was significantly greater at a higher temperature (*P* < 0.0001, *F*_2,83_ = 11.66, two-way ANOVA; Shapiro–Wilk’s* W*-test revealed normality of the distribution at *P* = 0.0802). These statistics did not, however, reveal any interaction between temperature and prey distribution, even though the increase in capture rate at higher temperatures evidently stems from the increased speed of a foraging fish (Table 2). However, its greater value in the patchy prey distribution may also be related to the ability of fish to be flexible enough to gain greater speed when outside the patch, and then slow down when within the patch of prey. The average swimming speed of foraging fish was slightly but significantly influenced by temperature (two-way ANOVA, *P* = 0.0302, *F*_2,982_ = 3.5), and strongly influenced by the position within or outside of the patch (*P* = 0.0001, *F*_2,982_ = 40.1). The post hoc test revealed greater speed in the tanks with a low density of prey than in the tank with the patch of prey, and at 26 °C than 16 °C. No effect of temperature was found on speed in the homogeneous section. The speed-dependent time a fish needed to arrive at a patch of prey and the decision to stay in the patch after its first encounter or to continue to search for a more profitable site were both important factors involved in securing capture rates equivalent to those accomplished by fish grouping in the high-prey-density tank within the first two minutes of prey delivery into the system (Figs. 3, 4). Note that the number of fish leaving the high-prey-density tank within the first 2 min of prey delivery (Fig. 3d) was not much smaller than the number of fish entering this tank (Fig. 3c), suggesting that many fish lost the opportunity for high capture rates within the first 2 min if returning to the high-prey-density tank when prey density was already much reduced (Fig. 4).

As a result of an apparent increase in foraging activity (combined with the testing of different tanks), the number of fish rapidly increased in the high-prey-density tank in the section with the patchy prey distribution, to quickly attain 40–70 % of all fish in the entire section. This congregation occurred more rapidly with temperature increase (Fig. 3a), but there was still a high proportion of fish foraging within the remaining nine tanks of the section, as the number of fish entering the high-prey-density tank (Fig. 3c) was roughly counterbalanced by the number of fish leaving the tank (Fig. 3d). The number of fish exiting the high-density tank was extremely variable, but was already very high in the first few minutes of each feeding session, particularly at the highest temperature (Fig. 3d). However, the departures (Fig. 3d) were more than compensated by the number of fish entering the tank containing the patch of prey (Fig. 3c). The departure rate (the ratio of the number of fish exiting compared to the number in the tank) gradually declined within the first 5 min of each feeding session, but there were still many fish (50 % on average) foraging in other parts of the section where prey density levels were more than an order of magnitude lower than in the patch. An apparent increase in the number of fish leaving the high-density tank was observed from the time when the density of prey had been reduced by half: 5, 3.5, and 2 min since prey introduction at, respectively, 16, 21 and 26 °C (Fig. 4a).

### Variability in capture rate in patchy prey distribution

Variability in per capita capture rates during the feeding sessions was orders of magnitude greater in the patchy distribution section than in the homogeneous section of the experimental system (Table 3). The difference was most apparent in the first 2 min of the feeding session (Fig. 1c), when the high initial density of prey (Fig. 4a) offered extremely high individual capture rates to the few resident fish and first arrivals to the high-prey-density tank. The prey density of 6–32 individuals L^−1^ gave a short-lasting theoretical chance of capturing prey at rates of up to 40, 50 and 60 prey min^−1^ at the temperatures of 16, 21 and 26 °C, respectively, i.e., 1 prey s^−1^ at the highest temperature (Fig. 4b, c). The initial capture rate (during the first 2 min of the feeding sessions) was strongly affected by the site (*P* < 0.0001, *F*_2,102_ = 892.8), and slightly affected by the temperature (*P* < 0.0011, *F*_2,102_ = 7.3). The high initial variability in prey-density-dependent mean capture rate of the experimental rudd was gradually reduced due to the rapid decline in the number of prey in the patch (Fig. 4a), which could be further expanded due to the increasing number of fish in the high-prey-density tank (Fig. 3a, b).

**Table 3:** Variability (coefficient of variation, % of the mean) of the capture rate of experimental fish throughout the experiments’ duration in the high-prey-density tank (A) and in the nine remaining tanks (B) of the section with patchy prey, and (in italic) in the entire section with patchy prey (A and B) and the entire section with homogeneous distribution (HD) of *Artemia* prey at each of the three temperatures: 16, 21 and 26 °C

Temperature °C	A	B	A and B	HD
First 2 min				
16	262.7	0.2	*2261.9*	*12.6*
21	336.0	4.1	*2340.5*	*41.8*
26	395.1	1.6	*2731.0*	*35.0*
First 10 min				
16	1127.2	1.7	*1060.8*	*76.7*
21	1349.6	20.8	*1350.0*	*191.2*
26	1355.0	8.2	*1621.1*	*153.7*

Data in italics expose great difference in variability between the two sections

### Effect of temperature

**Fig. 5:**
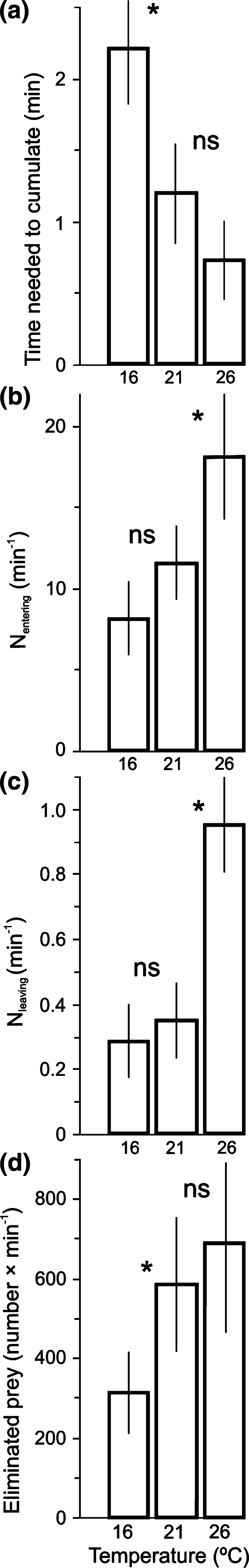
The rates (mean ± 1 SE) of fish aggregating in, entering and exiting the high-prey-density tank in the section with the patchy prey distribution and the initial effect of fish on prey density at temperatures of 16 °C (*n* = 9), 21 °C (*n* = 16) and 26 °C (*n* = 12). **a** The time needed for 15 % of the total number of fish in the section to congregate in the high-prey-density tank (assessed from the data in Fig. 3a), **b** the number of fish entering the high-prey-density tank in the first minute of the feeding session (assessed from the data in Fig. 3c), **c** the number of fish exiting the high-prey-density tank in the first minute of the feeding session (assessed from the data in Fig. 3d), **d** the number of prey eliminated from the high-prey-density tank in the first 2 min of the feeding session (assessed from the data in Fig. 4c)

Regardless of prey distribution, fish fed faster at higher temperatures (Figs. 2, 4). Similarly, fish cruised faster at high temperatures everywhere except in the high-prey-density tank of the patchy treatment (Table 2). Elevated temperatures also increased the initial capture rate in the high-prey-density tank (Fig. 4c). However, the rate increases with higher temperature were substantially less than the typical doubling with a 10 °C increase (*Q*_10_ = 2) characteristic of physiological phenomena (Fig. 5).

A two-way ANOVA test revealed that the effect of temperature was significant for each of the comparisons: the capture rate was greater at higher temperatures at each of the three initial prey concentrations (Figs. 2, 4b). The difference was greatest in the first 2 min following prey delivery (Fig. 4c); the cruising speed was greater at the higher temperature, the foraging fish being faster at higher temperatures everywhere except for the high-prey-density tank of the section with patchy prey distribution. However, the rates of temperature-induced increase were substantially less than the typical doubling with a 10 °C increase (*Q*_10_ = 2) characteristic of physiological phenomena. The effect of temperature was much greater when comparing the more complex fish behaviours represented in the following data: (1) the time needed for 15, 20 and 25 % of the total number of fish within the section with the patchy prey distribution to assemble in the high-prey-density tank (Fig. 5a; Table 4), (2) the number of fish entering (Fig. 5b) and exiting (Fig. 5c) the high-prey-density tank, and (3) the undetected ability to learn and remember how to find the patch of prey after several weeks of feeding in a homogeneous prey distribution situation. For the full temperature range of 16–26 °C, *Q*_10_ values of 3.01 and 2.44 were found for the time needed by 15 and 25 %, respectively, of fish in the section to assemble in the high-prey-density tank. The *Q*_10_ was even higher when calculated for the 16–21 °C range (*Q*_10_ = 3.42 and 4.00; Table 4). The *Q*_10_ was also much higher than two for the number of fish leaving the high-prey-density tank in the first minutes of the feeding session (Fig. 5c). One-way ANOVA revealed a strong effect of temperature in the time needed for 15, 20 and 25 % of the total number of fish within the section with the patchy prey distribution to assemble in the high-prey-density tank at *P* < 0.0001 (*F*_2,39_ > 12.26), with the significant difference between 16 °C and each of the two remaining temperatures (Fig. 5a). One-way ANOVA also revealed an increase in the number of fish entering the high-prey-density tank in the 1st and 2nd minutes after prey delivery to the experimental system at *P* < 0.0017 (*F*_2,93_ > 6.83), the difference significant only for 26 °C compared to 16 and 21 °C (post hoc test; Fig. 5b). The number of fish leaving the high-prey-density tank in the 1st and 2nd minutes following prey delivery was significantly greater in higher temperatures at *P* < 0.0002 (*F*_2,93_ > 9.43), with a significant difference only at 26 °C compared to 16 and 21 °C (post hoc test; Fig. 5c). The increase in the number of eliminated prey with temperature (Fig. 5d) was found to be significant at *P* = 0.0112 (*F*_2,42_ > 4.43), with the post hoc test indicating a difference only between 21 and 26 °C.

**Table 4:** *Q*_10_ values for the entire temperature range (16–26 °C) and two sub-ranges (16–21 and 21–26 °C) for the mean values of mobility and capture rate of fish foraging for *Artemia* prey in the sections with patchy and homogeneous prey distribution at three temperatures (16, 21 and 26 °C)

Parameter	16–26 °C	16–21 °C	21–26 °C
Speed of fish when cruising			
At heterogeneous prey distribution within the patch	*1.24*	1.02	1.51
At heterogeneous prey distribution outside the patch	*1.24*	1.47	1.24
At homogeneous prey distribution	*1.15*	1.18	1.11
Time needed to assemble in the patch (Fig. 5a)			
By 15 % of fish	*3.01*	3.42	2.66
By 20 % of fish	*2.41*	3.90	2.80
By 25 % of fish	*2.44*	4.00	1.53
No. fish entering high prey-density tank (Fig. 5b)			
In the 1st minute of the feeding session	*2.23*	2.03	2.46
In the first 2 min of the feeding session	*2.10*	2.51	1.76
No. fish exiting high prey-density tank (Fig. 5c)			
In the first minute of the feeding session	*3.33*	1.50	7.46
In the first 2 min of the feeding session	*3.13*	2.31	4.26
Number of prey eliminated from high-prey-density tank in first 2 min (Fig. 5d)	*2.21*	3.48	1.40
Initial capture rate (prey min^−1^; Fig. 4b)			
Of heterogeneous prey within the patch	*1.25*	1.24	1.27
Of heterogeneous prey outside the patch	*1.43*	2.04	1.33
Of homogeneous prey	*1.41*	1.25	1.66
Capture rate (prey min^−1^; Fig. 2) of fish			
At heterogeneous prey distribution	*1.69*	1.22	1.51
At homogeneous prey distribution	*1.78*	1.13	1.57

Statistically significant differences (*P* < 0.001) indicated by italics

## Discussion

### Higher food intake and more variable individual capture rates when prey patchily distributed

The results of this study provide experimental confirmation of the hypothesis that patchy prey distribution permits more efficient feeding by a predator than a homogeneous distribution of the same quantity of prey. The difference in individual capture rates for patchy and homogeneous prey was found to be highly consistent at each temperature, but surprisingly did not decline with increasing temperature in response to the reduced costs of foraging as expected (see “Temperature dependence of fish foraging behaviour, top–down cascades, and the global-warming perspective” section below). The possibility of higher food intake in an environment where food is patchily distributed does not, however, necessarily mean an overall greater individual fitness. The costs of seeking more rewarding patches of prey might be as high as the additional benefits gained from feeding within the patches due to the energy and time invested in sampling alternate sites for food abundance (McNamara and Houston 1985, 1987), long-term memorising (Milinski 1994), and learning to rely on the memory of conspecifics (Webster and Hart 2006). It does mean, however, that patchy prey distribution may offer predators the opportunity of far greater capture rates when encountering a sudden appearance of a patch of prey, such as that reported for *Daphnia* being concentrated in the upwelling regions of Langmuir spirals due to their downward movement against the current to escape the high light intensity at the surface (George and Edwards 1973). The learnt ability, or just good luck, to find such a short-lasting prey aggregation before it has been discovered and demolished by others, offers the fortunate fish a capture rate that might be an order of magnitude higher than that of a few minutes later, as is clearly illustrated by our experimental results (Fig. 4b). Such a prospect might possibly be the reason why planktivorous fish are usually on the move, seeking patches of prey, and often ignoring small-bodied prey encountered when searching for more profitable sites, where greatly differentiated individual capture rates stemming from priority of arrival would ensure an increased variability among individuals, hence promoting individual differences within a fish population. The mobility of fish may be the reason why as distinct a patch distribution of zooplankton prey as that created in the experimental system of this study might be difficult to find in natural systems, even though it might be continually created by wind-driven surface turbulence (Folt and Burns 1999; Kerckhove et al. 2015). Patchy prey distribution in space may also have an impact on juvenile survival (Lasker 1975) when food shortage may become as much of a hard-selection factor as the risk to predation, with less fortunate individuals unable to find a patch of prey before it has been reduced by more fortunate, faster or smarter conspecifics, with the suitable proportions of time invested in low-speed ranging or harvesting, and high-speed searching for a more profitable site (Coughlin et al. 1992; Gliwicz et al. 2013).

### IFD and the MVT, or something else?

The concept of the IFD has usually been employed in studies with a long-term perspective of an evolutionary stable strategy to explain the final, equilibrium distribution of conspecifics within the gradient of a continuous input of resources (Tregenza 1995) that would allow each individual to travel to and stay in the place where its gain would be the highest. However, the IFD concept seems to be also applicable to the process of fast numerical and functional responses of a typical predator-harvester to an appearance of patchy distribution of its tiny prey, as sudden as that taking place in our experimental set up. Such distribution remains in a continuous flux resulting from the constant arrivals and constant departures of the predators to and from the site with the patch of prey (Fig. 3), but for each moment of time, e.g. for a minute, it might be regarded as an outcome of each individual’s tendency to continuously seek a more food-sufficient site. This notion is supported by the high number of fish leaving the high-prey-density tank after the first minutes of each feeding session, despite the prey density still being much higher than in the neighbouring region. Neither the earlier observations of rudd behaviour (Maszczyk et al. 2014), nor the video recording from the two experimental sections have ever suggested that the permanent appearance of fish in the other low-prey-density locations was due to interference competition resulting from either passive competition or aggressive behaviour in the high-prey-density tank that would further increase with the arrival of new individuals into the patch of prey. This is why the cause may instead stem from perceptual constraints such as weak memory, insufficient knowledge of the pattern of prey distribution, and an insufficient ability to accurately assess resource distribution (Abrahams 1986). Moreover, to ensure that the food intake was above the anticipated average over the entire habitat (Charnov 1976), typical planktivorous fish, such as rudd or roach, would eventually have to move on in search of the next patch of prey, particularly when an increasing number of conspecifics would result in rapid prey depletion at the present site (Gliwicz et al. 2013). This preference for continually searching for a more profitable site is strengthened as soon as the density of prey in the patch has been reduced and capture rates declined. Such behaviour supports the MVT rather than IFD theorem, with foraging fish becoming more prone to the risk of the site being overexploited towards the end of the feeding session, when the number of fish leaving the high-prey-density tank increases. Until this point, however, the subsequent minutes show that the proportion of fish foraging within the high-density tank shifts in accordance with the expectations based on the simplest IFD model of Sutherland (1983), or Abrahams’ (1986) notion of perceptual constraints, when each fish is supplemented with the time needed to sample the prey distribution in the section with patchy prey before it eventually decides to stay in the high-prey-density tank.

Intuitively, it might be guessed that in the absence of competition and perceptual constraints, the highest capture rate of each experimental fish would be secured if they all congregated within the patch at the same time and remained within it as long as the prey density was higher there than in the neighbouring region. Instead, the experiments revealed that: (1) the time to congregate was longer than expected, (2) the proportion of fish in the patch of prey was only marginally higher than 50 % of their total number in the section, and (3) even within the first 5 min of the feeding session with this proportion’s upsurge, fish were not only entering, but also leaving the patch of prey for other sites with prey densities much lower than that still within the patch.

The results of this study demonstrate that the foraging behaviour of planktivorous fish is in agreement with predictions made using the IFD and MVT approaches: a foraging fish would not necessarily stay within the patch of prey for all the time until the patch has been overexploited, since it evidently has to check from time to time whether another patch has appeared in the neighbourhood, as assumed in the model of Gliwicz et al. (2013). However, it comes back to the patch again when it has learnt that no other patches are available in the system. This may not happen in the field or in a system of greater capacity, when returning to the same patch would be difficult, and the fit to the model predictions could be even better than those at 21 °C according to the simplest IFD model of Sutherland (1983).

The results of this study also show that the optimal exploitation of patches of zooplankton prey leads to their rapid annihilation. This occurs as a result of the combined effects of the predator’s functional response [an increase in the capture rate up to the highest values ever observed in planktivorous fish in laboratory and field studies of 0.8 prey s^−1^ (Bartosiewicz and Gliwicz 2011)] and the predator’s rapid numerical response in space, when the decline in prey concentration and interference within the overexploited patch of prey may encourage fish to swim faster to compensate for the reduced encounter rate, thus increasing the probability of mechanical interference with one another (Persson 1986; Maszczyk et al. 2014). As a consequence, the role of aggregating in zooplankton as an antipredation defence against fish predation (Pijanowska and Kowalczewski 1997) becomes questionable, and this is the point where the results of this study meet the theoretical approach focused on the stabilizing role of predators in their top–down regulation of prey density and distribution in space and time (Morozov et al. 2011). As in Morozov et al.’s (2011) model, and also in our previous experimental studies (Gliwicz et al. 2013), the mechanisms of optimal prey exploitation by the rapid prey-density-dependent functional and numerical responses in space lead rapidly to homogeneity in prey density as soon as the local patch of prey has been overexploited and the predators have been attracted to more rewarding sites. Morozov et al.’s (2011) approach does not need any implicit assumption that patches of resources are discrete (as assumed in the MVT approach), or that resources are constantly produced at, or imported to, different sites in different quantities at a constant rate (as assumed in the IFD continuous and interference models). This may be why focusing on the stabilizing role of predator dispersal and the aggregation of prey population densities at different sites could promote a better understanding of the reasons why zooplankton patches are rather a short-lasting phenomenon in the field, particularly in well-illuminated waters, and in spite of the never-ending forces of water currents (George and Edwards 1973), as well as the strong biological drivers (Folt and Burns 1999) that lead to patch formation.

### Temperature dependence of fish foraging behaviour, top–down cascades, and the global-warming perspective

Post-capture accelerations and other fast starts by fish are believed to demand an order of magnitude more energy than swimming in one direction at constant speed (Domenici and Blake 1997; Tang et al. 2000). Therefore, the decision made by a foraging fish to slow down or not to slow down to capture an encountered prey item is likely to depend on whether or not the energy gain would be higher than the combined costs of capture and post-capture acceleration (Gliwicz et al. 2013). Greater speed applied at low prey-density levels would also increase prey-size selectivity (Maszczyk and Gliwicz 2014). However, since water viscosity declines with rising temperature, energy requirements may be greatly reduced as the temperature increases, particularly in the case of small fish, such as larval Atlantic haddock (*Melanogrammus aeglefinus)* (Hunt von Herbing and Keating 2003) or experimental rudd of 1 g fresh mass (used in this study), since ‘viscous forces may have more pronounced effects on small fish’ such as goldfish (*Carassius auratus*, Johnson et al. 1998) than on 150-g fresh mass sea bass (*Dicentrarchus labrax*), in which the ‘net cost of transport at a given speed was not influenced by the elevation of the water temperature’ (Claireaux et al. 2006) as expected in larger fish (Hein and Keirsted 2012). This might allow capture rates in a small fish such as juvenile rudd to be greater than expected from the *Q*_10_ = 2 assumption, i.e. that the metabolic rate of fish is doubled as the temperature increases by 10 °C.

Surprisingly, neither the mean nor the maximum capture rates recorded in the present study revealed any cases of *Q*_10_ > 2, despite the high variability detected at each temperature. In fact, all calculated values of *Q*_10_ were lower than 2. The only exception was the number of prey eliminated from the high-prey-density tank in the first few minutes of each feeding session (*Q*_10_ = 2.21), but this was probably the effect of the rapid accumulation of fish in the patch of prey rather than the increased capture rate (*Q*_10_ < 2), which is supported by *Q*_10_ > 2.4 observed in the time needed for fish to assemble in the patch, and by *Q*_10_ > 2.1 observed for the mobility of fish entering and leaving the high-prey-density tank (Table 4). These findings were inconsistent with earlier work, where the data has allowed *Q*_10_ values to be calculated at much higher levels, exceeding 3, when estimated from the capture rate data of Wurtsbaugh and Cech (1983) for mosquito fish also feeding on *Artemianauplii* in the range of 15–25 °C, the data of Persson (1986) on roach feeding on zooplankton in the range of 12–21 °C, or the data of Bergman (1987) on perch fed phantom midge larvae within the range of 8–18 °C. The *Q*_10_ values of capture rate presented in this paper could possibly be much higher if not obscured by the effect of the fast increase in the number of fish arriving at the patch, which did not really have a chance to contribute to the capture rate estimated for all of the fish present in the tank with the patch of prey at the end of this period. Moreover, this was particularly true at 26 °C, when the initial capture rates could be much higher than those estimated for the first 2-min period, when the values had to be divided by a twice greater number of fish than that at the initial time.

The strength of the effect of temperature increase on the importance of the top–down effect of fish predation on zooplankton density and body size could be less important in the functional responses of individual fish to higher temperatures than the rapidity of fish numerical responses in space to patchy prey distribution (Gliwicz et al. 2013), which also could be observed in the experiments presented in this paper. The greatest sensitivity to a temperature increase, reflected by *Q*_10_ > 3, was found in the number of fish departing the high-prey-density tank (or merely leaving briefly to check the proficiency of the neighbouring tanks), and in the time needed for the majority of fish in the system to assemble at the most rewarding site. Such a large increase in *Q*_10_ could in part be due to the reduced obligatory investment for post-capture accelerations at higher temperatures (Gliwicz et al. 2013) or due to the reduced energy required at higher temperatures for the necessary process of warming the neural system of fish, particularly their brains and visual sensors (Fritsches et al. 2005; Sepulveda et al. 2007) that are vital in the search for tiny zooplankton prey in reduced light intensities at the time of the dusk or dawn anti-predation window (Clark and Levy 1988). Such a large increase in *Q*_10_ could be also due to more efficient learning and memorizing the location of the closest and most rewarding site, and in deciding whether their own long-term memory information or social information cues should be used in the search (Webster and Hart 2006). Such speculations make the high *Q*_10_ values for fish mobility, in their never-ending search for more rewarding sites, an important factor underlying the differences in individual fitness that is probably reinforced by the everyday race to locate the most rewarding site before it is found by others. The earlier observations of high *Q*_10_ values for the foraging efficiency of fish combined with the results of the present study support the hypothesis that the top–down effect of planktivorous fish on zooplankton may be strengthened by global warming. The increase in temperature should permit faster learning and better decisions by foraging planktivorous fish. The reduced water viscosity at higher temperatures also lowers the cost of post-capture accelerations and allows small-bodied fish to persist in their foraging efforts despite reduced zooplankton abundance, decreased body size in cladoceran populations, and the absence of large-bodied cladocerans (such as the most-vulnerable *Daphnia* species). This may be further enhanced by the reduced fear of predation by large piscivorous fish, which do not have the benefit of reduced viscosity as ‘rising water temperature may increase the energetic cost of routine swimming behaviours’, as recently suggested by Hein and Keirsted (2012). This hypothesis offers a complementary explanation of latitudinal patterns in cladoceran size distribution [*Daphnia* body size declining from the high latitudes to the equator (Gillooly and Dodson 2000)], and supports the notion that the top–down cascade of fish predation is an important factor in promoting algal and cyanobacterial blooms through reduced grazing when at elevated temperatures. Blooms may indeed ‘like it hot’ as claimed by Paerl and Huisman (2008), but not exclusively because of the relaxed bottom-up limitation at higher temperatures in the context of global warming as they suggest. The findings of the present study imply that small planktivorous fish also ‘like it hot’ when reduced viscosity allows the persistence of foraging efforts despite reduced zooplankton abundance, decreased body size in cladoceran populations, and the absence of large-bodied *Daphnia*, which would otherwise control algal and cyanobacterial populations.
